# Improved Pressure Sensing Performance of Self-Powered Electrochemical Pressure Sensor Using a Simple Electrode Coplanar Structure

**DOI:** 10.3390/s26020699

**Published:** 2026-01-21

**Authors:** Yixue Han, Zaihua Duan, Yi Wang, Weidong Chen, Di Liu, Zhen Yuan, Yadong Jiang, Huiling Tai

**Affiliations:** State Key Laboratory of Electronic Thin Films and Integrated Devices, School of Optoelectronic Science and Engineering, University of Electronic Science and Technology of China (UESTC), Chengdu 611731, China

**Keywords:** electrochemical pressure sensor, coplanar electrode structure, self-powered, respiratory state recognition, machine learning

## Abstract

In recent years, electrochemical pressure (ECP) sensors with self-powered and both dynamic and static pressure detection capabilities have received widespread attention. To improve pressure sensing performances while reducing the thickness of conventional sandwich structure ECP sensors, we propose an ECP sensor with a simple electrode coplanar structure. Specifically, it consists of Cu/Zn foil electrodes and LiCl/polyvinyl alcohol (PVA) modified filter paper. Among them, the Cu/Zn coplanar electrodes are used for redox reactions, the LiCl provides conductive ions, and the PVA is used to provide a humid environment to promote the ionization and conduction of LiCl. The rough surface microstructure of the filter paper is used to enhance the pressure sensing performances of the sensor. The results show that the ECP sensor with an electrode coplanar structure can spontaneously output current in the pressure range of 0.4–100 kPa, with sensitivities of 0.273 kPa^−1^ (0.6–20 kPa) and 0.036 kPa^−1^ (20–100 kPa). Specifically, compared to ECP sensors with a sandwich structure, it has a wider response range and higher sensitivity. Through the current response, morphological characterizations, and redox reactions, the pressure sensing mechanism is elucidated. Furthermore, the proposed ECP sensor can be used for respiratory state recognition combined with machine learning. This research provides a new approach for developing a high-performance ECP sensor with a simple electrode coplanar structure.

## 1. Introduction

Flexible pressure sensors have shown great potential for motion monitoring, robot tactile perception, and health monitoring [1,2,3,4,5]. After years of development, flexible pressure sensors have evolved into various types, including resistance [6,7,8,9], capacitance [10,11,12], piezoelectricity [13,14,15], triboelectricity [16,17,18,19], ion gradient [20,21,22,23], and electrochemistry [24,25,26,27,28,29,30,31,32,33,34,35]. Among them, piezoelectric, triboelectric, ion gradient, and electrochemical pressure (ECP) sensors can spontaneously generate voltage or current and can be classified as self-powered pressure sensors [13,14,15,16,17,18,19,20,21,22,23,24,25,26,27,28,29,30,31,32,33,34,35]. Ionic gradient and ECP sensors can overcome the shortcomings of piezoelectric and triboelectric pressure sensors in detecting static pressure [20,21,22,23,24,25,26,27,28,29,30,31,32,33,34,35]. Compared to ion gradient pressure sensors, the ECP sensors have greater potential for power generation and have received a lot of attention in recent years [24,25,26,27,28,29,30,31,32,33,34,35].

The ECP sensors integrate electrochemical reactions and piezoresistive sensing mechanisms [24,25,26,27,28,29,30,31,32,33,34,35]. In order to improve the performances (mainly including pressure response range, sensitivity, repeatability, response/recovery time, and power generation capacity) of ECP sensors, many works focus on electrodes, electrolytes, and pressure sensing layers. Taking electrode materials as an example, researchers commonly utilize metallic materials such as Cu, Zn, Mg, and Al, as well as other materials like MnO_2_ and carbon, to construct electrode configurations with a sandwich architecture for various ECP sensors [24,25,26,27,28,29,30,31,32,33,34,35]. Among various electrolytes, LiCl has good ion conductivity and is commonly used in the preparation of EC sensors [36,37,38], including ECP sensors [27,32]. In order to achieve good pressure sensing response of ECP sensors, the microstructured materials are often used as pressure sensing layers, mainly including paper-based materials, hydrogel systems, porous polymer frameworks, carbon-based composites, and fiber–gel composite structures [24,25,26,27,28,29,30,31,32,33,34,35]. Among them, cellulose paper has an inherent microstructure and flexibility, as well as low cost and green environmental advantages, and is commonly used to construct various flexible pressure sensors [39,40,41], including ECP sensors [24,27,32]. The ECP sensors reported above all adopt a sandwich structure, where electrodes are located on both sides of the pressure-sensitive layer [24,25,26,27,28,29,30,31,32,33,34,35]. Although they achieved satisfactory pressure sensing and power generation performances through material design, the sandwich structure itself increases device thickness, which is not conducive to excellent flexibility. If the positive/negative electrodes are designed on the same side of the pressure sensing layer, it is expected to address the shortcomings of sandwich structure ECP sensors. In addition, the electrode coplanar structure ECP sensor can not only utilize the interface characteristics between the pressure sensing layer and the electrodes but also hope to improve its pressure sensing performance by utilizing the gap spacing between the positive and negative electrodes.

Based on the above discussion, this work introduces a novel coplanar structure ECP sensor that circumvents the limitations of conventional sandwich configuration. It uses LiCl/polyvinyl alcohol (PVA) (LP) filter paper as the pressure sensing layer, with the Cu positive electrode and Zn negative electrode located on the same side of the pressure sensing layer. The PVA matrix maintains a humid microenvironment to facilitate LiCl dissociation and ion transport, thereby improving overall ionic conductivity. The inherent microstructure of the filter paper is utilized to enhance pressure sensing performance. The resulting coplanar structure LP-ECP sensor demonstrates notable performances compared to the sandwich structure LP-ECP sensor. Combining mechanism analysis and application verification, this work provides new insights for the development of high-performance ECP sensors using a simple electrode coplanar structure.

## 2. Experimental Section

### 2.1. Materials

The Zn foil (thickness: ~60 μm, width: 6 mm) and Cu foil (thickness: ~65 μm, width: 6 mm) were procured from Milage Adhesive Co., Ltd., Shenzhen, China. Polyimide (PI) tape (thickness: ~55 μm) was obtained from Huijia Adhesive Products Co., Ltd., Shenzhen, China. Filter paper (thickness: ~210 μm) was purchased from Fuyang Beimu Pulp Co., Ltd., Fuyang, China. LiCl (analytical reagent grade) was acquired from Shanghai Aladdin Biochemical Technology Co., Ltd., Shanghai, China. PVA (K-90) was obtained from Sinopharm Chemical Reagent Co., Ltd., Shanghai, China. All raw materials were used directly without further purification treatment.

### 2.2. Preparation of the LP Pressure Sensing Layers

The preparation process of the LP pressure sensing layers is as follows: First, LiCl solutions with concentrations of 0, 0.5, 1, 1.5, 2, and 2.5 mol/L were prepared based on our previous research foundation [27,32]. Specifically, 0, 0.106, 0.212, 0.318, 0.424, and 0.531 g, respectively, of LiCl were dissolved in 5 mL deionized water. Subsequently, 4 g of PVA powder was mixed with 20 mL of deionized water and stirred at 80 °C for 30 min to obtain PVA solution. Finally, 2 mL of PVA solution was added to each of the above LiCl solutions, followed by vigorous stirring at 80 °C for 20 min to prepare the different LP electrolytes. It should be pointed out that the influence of LiCl concentration was studied while maintaining a constant PVA content. In addition, comparative studies were conducted using pure LiCl solution (2 mol/L based on subsequent pressure sensing performances) and pure PVA solution.

### 2.3. Fabrication of the LP-ECP Sensors

**Figure 1:**
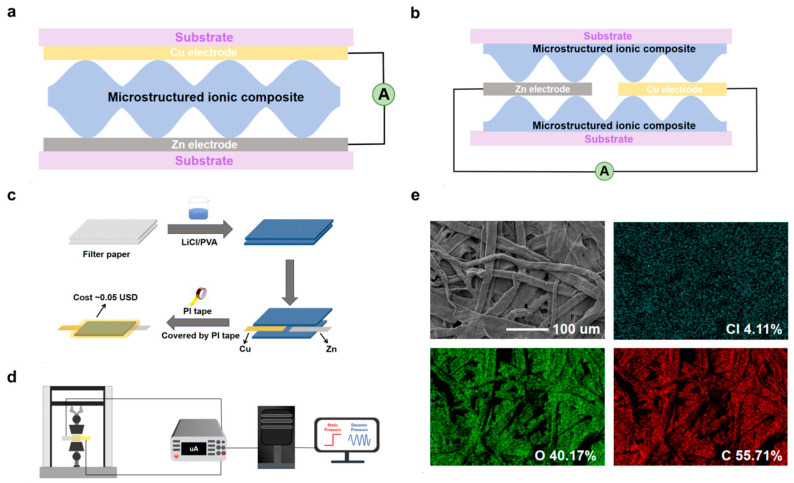
(**a**) Schematic diagram of the sandwich structure LP-ECP sensor. (**b**) Schematic diagram of the electrode coplanar LP-ECP sensor. (**c**) Fabrication process diagram of the electrode coplanar LP-ECP sensor. (**d**) Schematic diagram of the pressure sensing test system. (**e**) SEM image of the LP filter paper and the corresponding Cl, O, and C element mappings.

Figure 1a shows the schematic diagram of the conventional ECP sensor, with a sandwich structure of electrodes on opposite sides. In contrast, this work employed a simplified coplanar structure LP-ECP sensor (Figure 1b). Figure 1c illustrates the fabrication process of the electrode coplanar structure LP-ECP sensor. Cu and Zn foil electrodes were first cut into strips (5 cm length, 6 mm width). The filter paper was cut into small squares (1 cm × 1 cm) and then immersed in the different LP electrolytes and dried. The assembly process involves placing the LP electrolyte-loaded filter paper onto a PI tape substrate, followed by aligning the Cu and Zn electrodes with an electrode spacing of 3 mm on the filter paper and, finally, vertically stacking a second layer of LP electrolyte-loaded filter paper. In addition to the optimal electrode spacing of 3 mm, 1, 2, 4, and 5 mm were also tested. The LP-ECP sensor was finally encapsulated with PI tape. For comparison, the sandwich structure LP-ECP sensor has also been prepared using the LP electrolyte (2 mol/L LiCl) based on the pressure sensing performance of the optimized electrode coplanar structure LP-ECP sensor. Considering the cost of raw materials, the estimated cost per sensor is very low, approximately 0.05 USD.

### 2.4. Characterization and Measurement of the LP-ECP Sensors

The morphological characterization of the optimized LP filter paper (2 mol/L LiCl) was performed using scanning electron microscopy (SEM, GeminiSEM 300), with elemental distribution analyzed by energy-dispersive X-ray spectroscopy (EDS, Ultim Max, Oxford Instruments). Cross-sectional structural changes in the LP-ECP sensor under pressurized and non-pressurized states were observed via optical microscopy (Saikedigital). As illustrated in Figure 1d, different pressures were applied to the LP-ECP sensor using a universal testing machine (ZC-990B, error: ±0.5%), and the current response of the LP-ECP sensor was recorded using a digital multimeter (DMM 6500, Keithley) [27,32]. The pressure sensing performance test is conducted in a typical laboratory environment, with the temperature controlled by air conditioning at approximately 25 °C. The current response was defined as ∆I/I_0_, where I_0_ denotes the baseline current at zero pressure and ∆I represents the current variation under loading pressure. The sensitivity (S) of the LP-ECP sensor was calculated as S = δ(∆I/I_0_)/δP, where δP indicates the pressure change within the linear response region [42,43].

## 3. Results and Discussion

### 3.1. Characterization of Morphology and Element Distribution

Figure 1e shows the SEM image of the optimized LP filter paper (2 mol/L LiCl) and the corresponding Cl, O, and C element mappings, with a rough surface microstructure formed by irregularly interwoven fibers, which is beneficial for generating pressure sensing response [44,45,46,47,48]. Except for the Li element with low ionization energy, the other expected elements Cl, O, and C all appear in LP filter paper (Figure S1).

### 3.2. Performances of the LP-ECP Sensors

**Figure 2:**
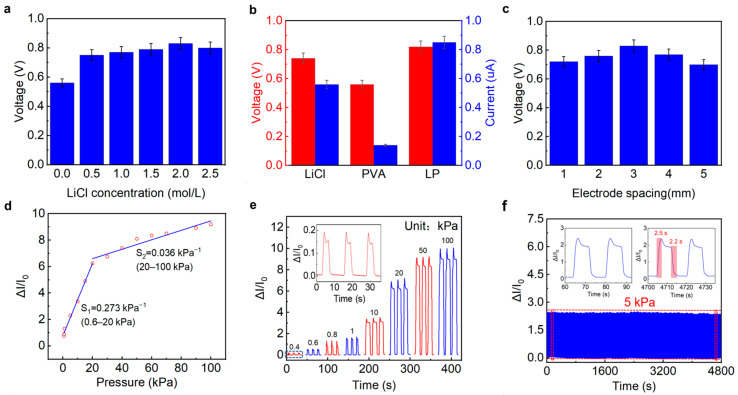
(**a**) Output voltages of the LP-ECP sensors with different LiCl concentrations under no pressure. (**b**) Open-circuit voltage and short-circuit current of the LP-ECP sensors with different electrolytes under no pressure. (**c**) Influence of electrode spacing on output voltage of the LP-ECP sensor. (**d**) Segmented linear fitting lines of current response versus pressure. (**e**) Current response and recovery curves under different pressures. (**f**) Response and recovery curves under 5 kPa over 300 cycles.

Considering that Li^+^ and Cl^−^ ions are the main carriers between the positive and negative electrodes, the influence of LiCl content on output voltage was studied first. As shown in Figure 2a, with the increase in LiCl concentration, the output voltage of the LP-ECP sensor first significantly increases and then slightly decreases under no pressure, which is consistent with previous reports [32]. This is because when the LiCl concentration is low, there are not enough ions involved in conduction; when the LiCl concentration is too high, the crystallization phenomenon of LiCl is not conducive to ion conduction. Therefore, it has the maximum output voltage when the LiCl concentration is 2 mol/L. Compared to the LP-ECP sensors based on pure LiCl and PVA, the LP-ECP (PVA and 2 mol/L LiCl) balances high output voltage (0.82 V) and short-circuit current (0.85 A) under no pressure (Figure 2b). In addition, the gap spacing between Cu and Zn electrodes can affect ion conduction, thereby affecting the output voltage of the LP-ECP sensor. As shown in Figure 2c, considering the output voltage value, the electrode spacing of 3 mm is optimal. The gap spacing between positive/negative electrodes can help to improve the contact area change between the upper and lower LP pressure sensing layers, thereby enhancing the pressure sensing response. However, it is not conducive to ion conduction between positive/negative electrodes. Therefore, a preferred 3 mm electrode gap width was obtained. Based on the above results, the following research focuses on the pressure sensing performance and application of the LP-ECP sensor (PVA and 2 mol/L LiCl electrolyte and 3 mm electrode gap spacing).

Given that current response provides more significant variation in ECP sensors, we evaluated the pressure sensing performances of LP-ECP sensor via output current [24,25,27,31,32,33]. As depicted in Figure 2d, the electrode coplanar LP-ECP sensor exhibits a broad detection range from 0.4 to 100 kPa. According to the segmented linear fitting results, the sensitivities are 0.273 kPa^−1^ (0.6–20 kPa) and 0.036 kPa^−1^ (20–100 kPa), respectively. Flexible pressure sensors typically exhibit segmented linear responses, with higher sensitivity in the low-pressure range than in the high-pressure range. This is mainly because at low pressure, the contact area between the pressure sensing layers rapidly increases, while at high pressure, the rapid increase in contact area slows down and gradually saturates, similar to previous reports on ECP sensors [24,25,26,27,28,29,30,31,32,33,34,35]. Correspondingly, Figure 2e shows the response and recovery curves of the LP-ECP sensor under different pressures, with a clear response to low pressure of 0.4 kPa. Compared to the sensitivity (0.012 kPa^−1^, 0.8–10 kPa) of the sandwich-structured ECP sensor (Figure S2), the electrode coplanar LP-ECP sensor has a wider detection range and higher sensitivity. Figure 2f shows 300 response and recovery curves under 5 kPa. In order to observe the details clearly, enlarged 300 cycle responses are shown in Figure S3, with a small relative standard deviation of 2.05%. However, due to the consumption of chemical reactions, it is difficult to avoid the degradation of the pressure sensing performance for the ECP sensor. In particular, when the ECP sensor is under high pressure, it will intensify the chemical reactions and material consumption. In order to obtain more sustainable pressure sensing performance, it is necessary to use the development path of high-performance batteries, such as seeking high-energy materials or adopting charging strategies [35]. According to the inset of Figure 2f, the response and recovery times of the electrode coplanar LP-ECP sensor are 2.5 and 2.2 s, respectively. It should be noted that the response and recovery times of the pressure sensor are closely related to the movement speed of the pressure probe [39,40,41]. For example, by quickly tapping the LP-ECP sensor using a finger, it can achieve shorter response and recovery times (0.27 and 0.75 s), as shown in Figure S4. It should be noted that the ECP sensors are still in the early stages of research, and this study only tested some indicators (such as pressure response range, sensitivity, repeatability, and response/recovery time). The tested pressure sensing indicators are not yet complete, and as research progresses, it is necessary to refer to the relatively mature research on piezoresistive sensors [49,50,51,52].

**Figure 3:**
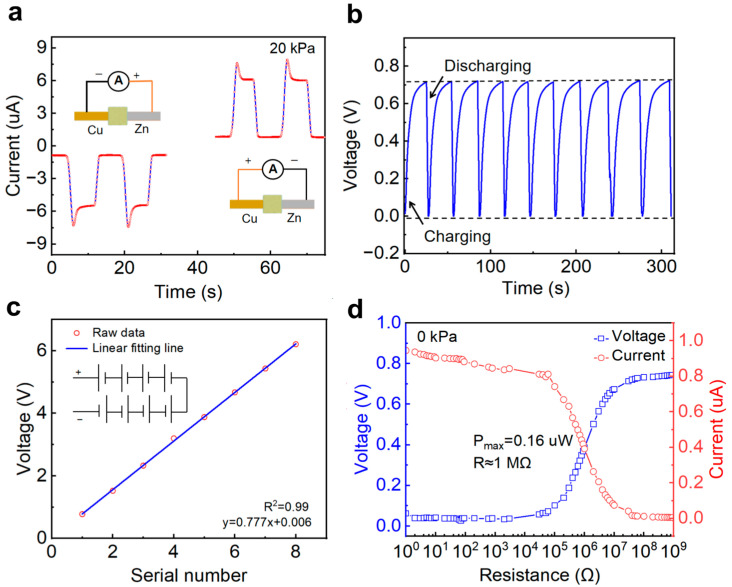
(**a**) Current response curves of the LP-ECP sensor with positive/negative connections under 20 kPa. (**b**) Voltage curve of a commercial capacitor (470 μF) during charging using the LP-ECP sensor. (**c**) Output voltage versus number of the LP-ECP sensor connected in series under no pressure. (**d**) Output voltage and current curves of the LP-ECP sensor under different loading resistances under no pressure.

Figure 3 evaluates the power generation performances of the LP-ECP sensor. By connecting the Cu positive and Zn negative electrodes, the results show that the LP-ECP sensor has the same positive and negative polarity as a battery (Figure 3a) [27,53]. From the charge–discharge curves in Figure 3b, the LP-ECP sensor can be used as a power source to the charge capacitor. Considering that the output voltage of a single LP-ECP sensor is very small, connecting multiple LP-ECP sensors in a series can increase the output voltage (Figure 3c). According to the voltage and current test circuits with different loading resistances (Figure S5), the maximum load power of the LP-ECP sensor can be obtained under no pressure. As shown in Figure 3d, with the load resistance increases, the load voltage gradually increases while the output current gradually decreases. When the load voltage curve intersects with the current curve (i.e., when the load resistance is equal to the internal resistance of the LP-ECP sensor), the maximum load power of 0.16 W can be obtained. Although the LP-ECP sensor can be used for power generation and capacitor charging, it cannot obtain pressure sensing signal while generating electricity. According to Figure 3a, the LP-ECP sensor is expected to drive the microampere pointer to obtain a visualized pressure sensing signal without an external power supply according to our previous report [27].

### 3.3. Pressure Sensing and Power Generation Mechanisms

**Figure 4:**
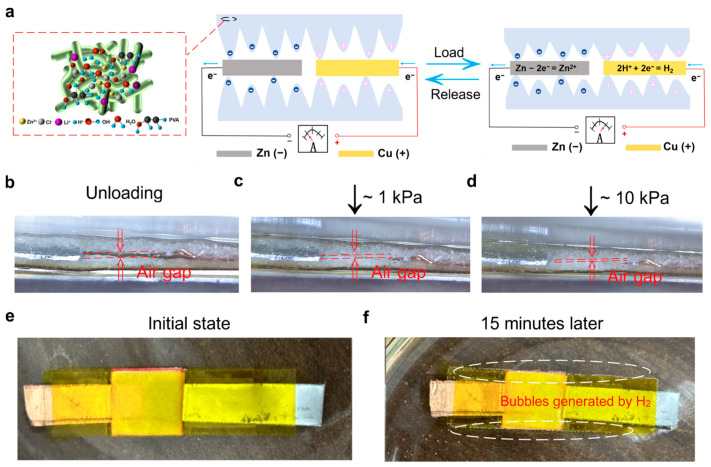
(**a**) Schematic diagram of the working mechanism of the LP-ECP sensor. (**b**–**d**) Optical photographs of the LP-ECP sensor under different pressures. Optical photographs of the LP-ECP sensor in water (**e**) at the initial stage and (**f**) after 15 min.

The pressure sensing and power generation capabilities of the electrode coplanar ECP sensor originate from coupled electrochemical reactions and pressure-induced microstructural changes. Figure 4a shows the schematic diagram of the working mechanism of the electrode coplanar LP-ECP sensor, which uses LP filter paper as the pressure sensing layer, with the Cu positive electrode and Zn negative electrode located on the same side of the LP filter paper pressure sensing layer. In this system, PVA maintains a humid environment that facilitates the dissociation of LiCl into Li^+^ and Cl^−^ ions, while generating H^+^ and OH^−^ ions due to the slight dissociation of water molecules (H_2_O → H^+^ + OH^−^) [24,25,26,27,28,29,30,31,32,33,34,35]. At the zinc negative electrode, an oxidation reaction occurs when zinc atoms lose electrons and convert to Zn^2+^ (Zn − 2e^−^ → Zn^2+^). At the Cu positive electrode, H^+^ ions capture electrons and convert them into H_2_, resulting in a reduction reaction (2H^+^ + 2e^−^ → H_2_). The oxidation and reduction reactions occurring at the electrodes establish the power generation behavior inherent to the ECP sensor [24,25,26,27,28,29,30,31,32,33,34,35]. The applied pressure will increase the interface contact area between the electrodes and the LP filter paper pressure sensing layer, resulting in the promotion of ion conduction and chemical reactions and the increase in output voltage (U). At the same time, the increased interface contact area between the electrodes and the LP filter paper pressure sensing layer will reduce the internal resistance (r) of the LP-ECP sensor, thereby significantly improving current response (I = U/r). This collaborative mechanism supports the self-powered pressure sensing response of the LP-ECP sensor.

To further elucidate the working mechanism, the cross-sectional structural changes were analyzed under different pressure conditions (Figure 4b–d). Under no pressure (Figure 4b), it can be seen that the interface contact between the LP filter paper pressure sensing layers is poor, limiting ion transport and resulting in high internal resistance of the sensor. Conversely, the applied pressure increases the interface contact area, enabling enhanced ion conduction and chemical reactions (Figure 4c,d). To further verify hydrogen evolution during the reduction reaction, the encapsulated LP-ECP sensor was immersed in water and monitored (Figure 4e,f). After soaking the LP-ECP sensor in water for 15 min, gas can be observed escaping around the sensor, confirming the cathodic reduction reaction that produces H_2_ [27].

### 3.4. Application Demonstration of the LP-ECP Sensors

**Figure 5:**
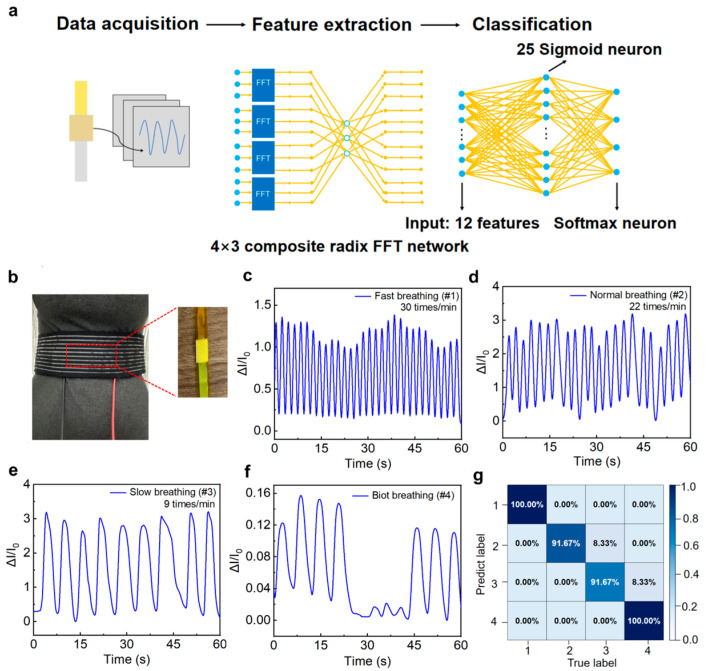
(**a**) Process flowchart of respiratory pattern classification based on machine learning algorithms. (**b**) Optical photograph of respiratory data collection using the LP-ECP sensor. Response curves for (**c**) fast, (**d**) normal, (**e**) slow, and (**f**) Biot’s respiration patterns. (**g**) Confusion matrix derived from BPNN model implementation for respiratory pattern recognition (label 1–4 corresponds to tachypnea, eupnea, bradypnea, and Biot’s respiration).

Respiratory rate, as an important physiological parameter, provides crucial information in clinical disease diagnosis and health monitoring [54,55,56]. Combined with machine learning algorithms, the LP-ECP sensor can achieve accurate respiratory patterns recognition, demonstrating the potential of intelligent health monitoring. To enable respiratory pattern classification, we employed a backpropagation neural network (BPNN), which is a widely adopted artificial neural network known for its robust nonlinear mapping and concise architecture, to process the respiratory data acquired by the LP-ECP sensor (Figure 5a). As shown in Figure 5b, the LP-ECP sensor is fixed on the waist belt to obtain respiratory rate through the expansion and contraction of the abdomen during breathing. To verify the respiratory pattern recognition function, different respiratory patterns were simulated and tested using the LP-ECP sensor, including fast, normal, slow, and Biot’s respiration (Figure 5c–f). For respiratory pattern recognition, the original data was first sampled at 5 Hz in accordance with the Nyquist theorem [27]. The dataset was divided into training (60%), validation (15%), and test sets (25%). The training/validation segmentation aims to capture different respiratory features and monitor overfitting during the training process. In addition, 25% of the data samples are allocated to the test set to evaluate the performance of the model on data that has not been trained. The signal was then transformed from the time to frequency domain via fast Fourier transform, followed by feature extraction using the BPNN algorithm and classification via a Softmax classifier. As shown in Figure S6, the cross-entropy loss function of the machine learning model exhibits closely aligned and low values on both the training and test sets. The resulting confusion matrix demonstrates a recognition accuracy exceeding 91.67% for all respiratory patterns (Figure 5g), indicating that the combination of the LP-ECP sensor and machine learning has potential application in respiratory pattern monitoring.

## 4. Conclusions

In summary, this work developed an electrode coplanar LP-ECP sensor based on LiCl/PVA electrolyte filter paper and Cu/Zn electrodes. Compared to a conventional sandwich structure ECP sensor, the electrode coplanar structure not only helps to reduce the thickness of the ECP sensor but also optimizes the LP-ECP sensor’s pressure sensing performances by adjusting the gap spacing between the Cu/Zn electrodes. It is noteworthy that the electrode coplanar LP-ECP sensor achieves an expanded detection range of 0.4 to 100 kPa while maintaining high sensitivity across both low-pressure and high-pressure regimes (0.273 kPa^−1^ at 0.6–20 kPa and 0.036 kPa^−1^ at 20–100 kPa). However, it must be admitted that the performance (such as sensitivity) of the electrode coplanar LP-ECP sensor cannot compare to many sandwich structure ECP sensors based on other material systems. Of course, on the basis of synchronously optimizing the material system, it is expected to improve the performance of electrode coplanar ECP sensors in the future. The underlying pressure sensing mechanism was elucidated through current response characteristics, cross-sectional morphological evolution, and redox reactions. Furthermore, the electrode coplanar LP-ECP sensor can be used for respiratory pattern recognition assisted with machine learning. Collectively, this study provides a scalable fabrication strategy for structural innovation in ECP sensors.

## Data Availability

The data of our study are available upon request.

## Supplementary Materials

The following supporting information can be downloaded at: https://www.mdpi.com/article/10.3390/s26020699/s1, Figure S1: Cl, O, and C element mappings of the LP filter paper; Figure S2: (a) Response and recovery curves of the sandwich-structured ECP sensor based on the same materials. (b) Response of the sandwich-structured ECP sensor at different pressures and the corresponding linear fitting line (0.8–10 kPa); Figure S3: Enlarged response and recovery curves under 5 kPa over 300 cycles (50 cycles per image); Figure S4: Response and recovery curves by quickly tapping the LP-ECP sensor using finger; Figure S5: (a) Voltage and (b) current test circuits with the different loading resistances; Figure S6: Loss changes of the machine learning model during training.
