# Supplementary material for: Improved Pressure Sensing Performance of Self-Powered Electrochemical Pressure Sensor Using a Simple Electrode Coplanar Structure
Source: Sensors (Basel). 2026 Jan 21;26(2):699. doi: 10.3390/s26020699 (PMC12845850; doi:10.3390/s26020699)
Supplement: Supplementary file 1 [file sensors-26-00699-s001.zip › sensors-4102440-supplementary.pdf]

Supplementary Material

Improved pressure sensing performance of Self-Powered Electrochemical Pressure Sensor Using a Simple Electrode Coplanar Structure

Yixue Han, Zaihua Duan*, Yi Wang, Weidong Chen, Di Liu, Zhen Yuan, Yadong Jiang and Huiling Tai*

State Key Laboratory of Electronic Thin Films and Integrated Devices, School of Optoelectronic Science and Engineering, University of Electronic Science and Technology of China (UESTC), Chengdu 611731, China

Corresponding authors: Zaihua Duan, Huiling Tai

E-mail: zaihuaduan@uestc.edu.cn, taitai1980@uestc.edu.cn

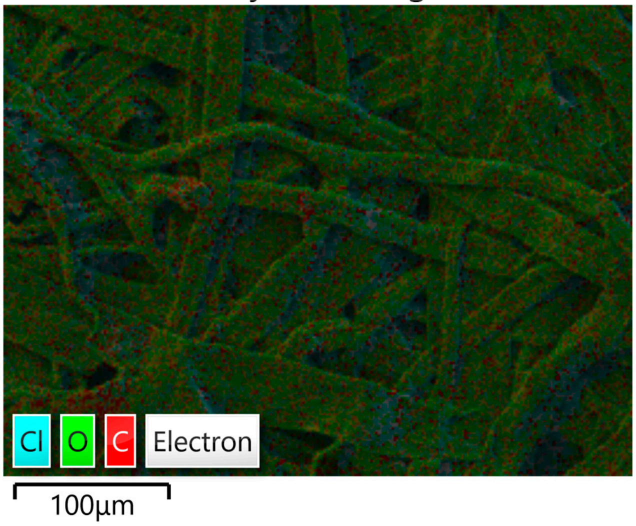

Figure S1. Cl, O, and C element mappings of the LP filter paper.

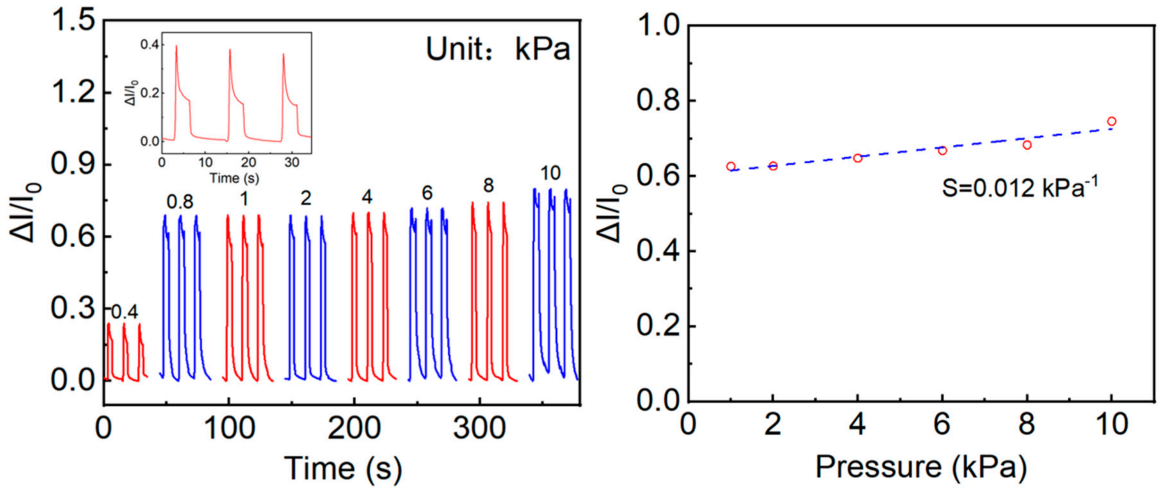

Figure S2. (a) Response and recovery curves of the sandwich-structured ECP sensor based on the same materials. (b) Response of the sandwich-structured ECP sensor at different pressures and the corresponding linear fitting line (0.8–10 kPa).

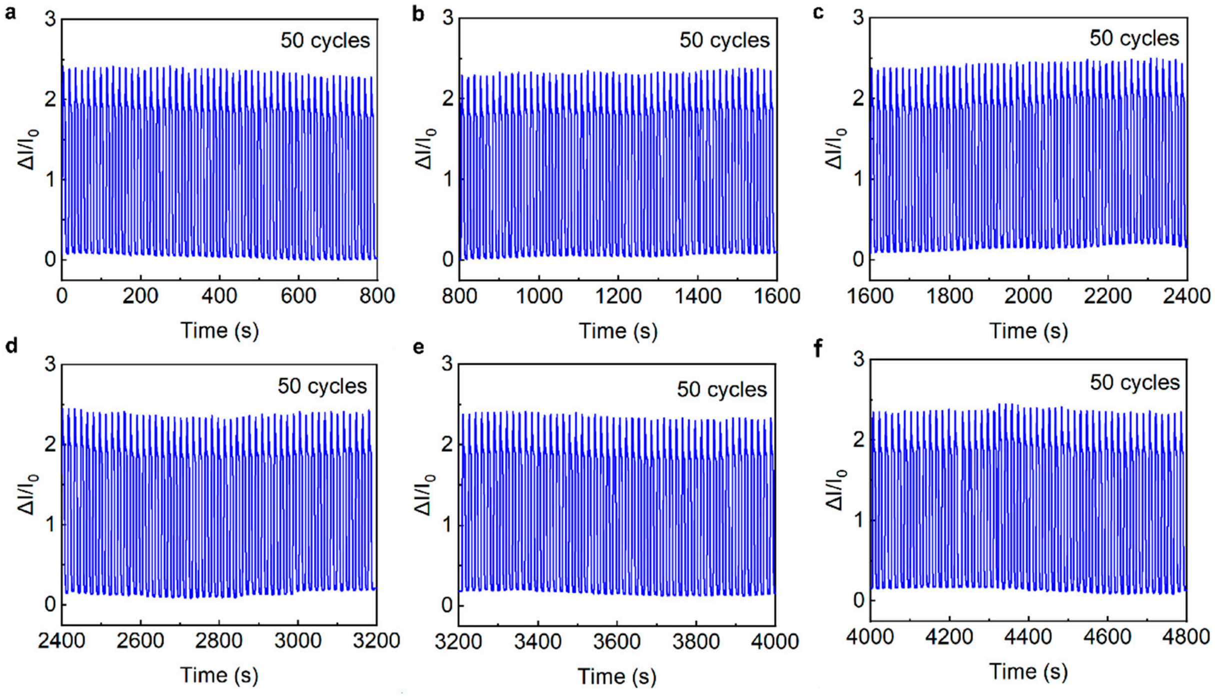

Figure S3. Enlarged response and recovery curves under 5 kPa over 300 cycles (50 cycles per image).

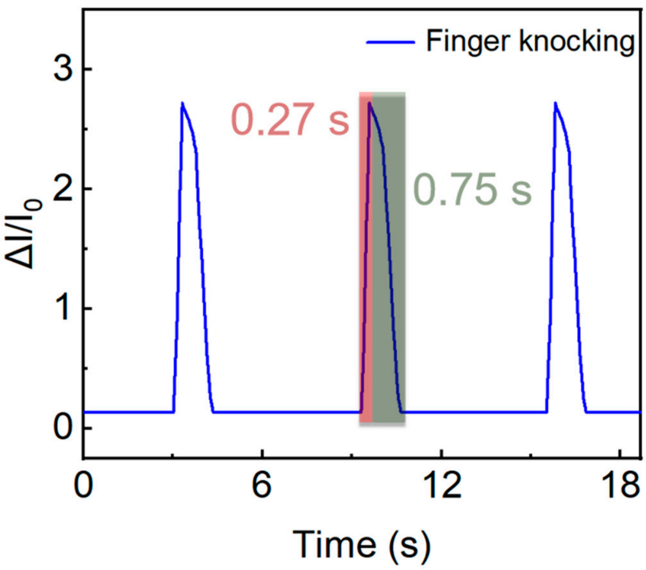

Figure S4. Response and recovery curves by quickly tapping the LP-ECP sensor using finger.

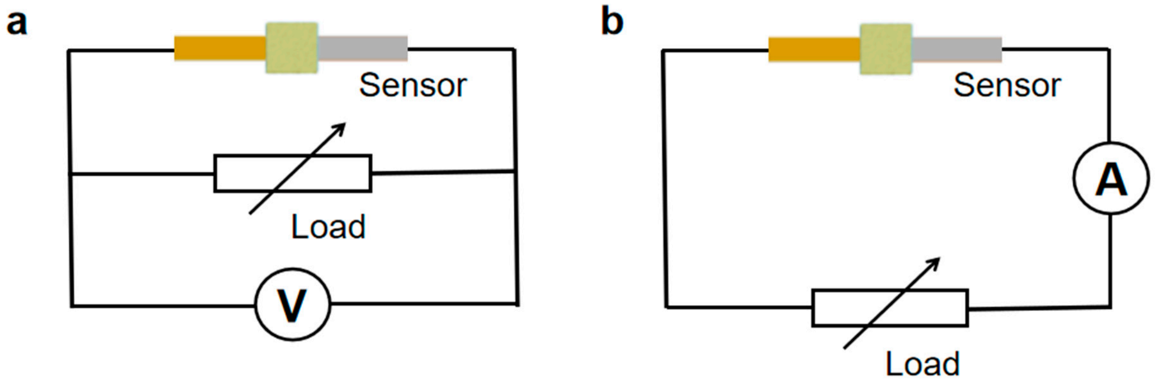

Figure S5. (a) Voltage and (b) current test circuits with the different loading resistances.

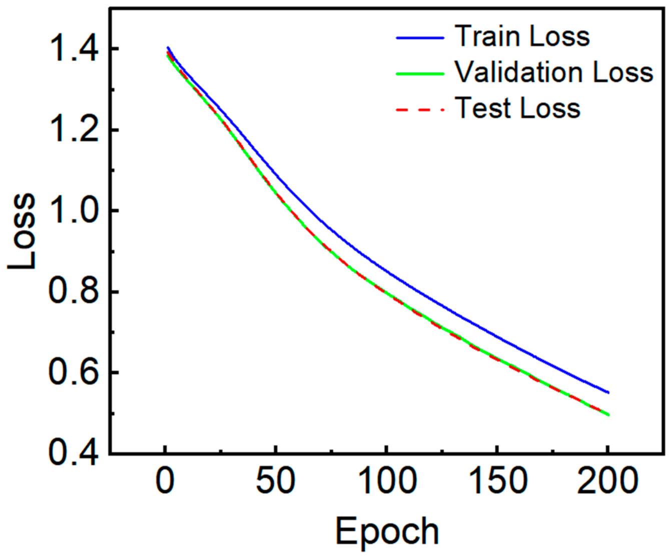

Figure S6. Loss changes of the machine learning model during training.
